# Supplementary material for: Loop Catalog: a comprehensive HiChIP database of human and mouse samples
Source: Genome Biol. 2025 Jun 20;26:175. doi: 10.1186/s13059-025-03615-5 (PMC12180236; doi:10.1186/s13059-025-03615-5)
Supplement: Supplementary file 1 — Additional file 1: Figures S1–S14. Supplementary Figures with their captions. [file 13059_2025_3615_MOESM1_ESM.pdf]

Fig. S1

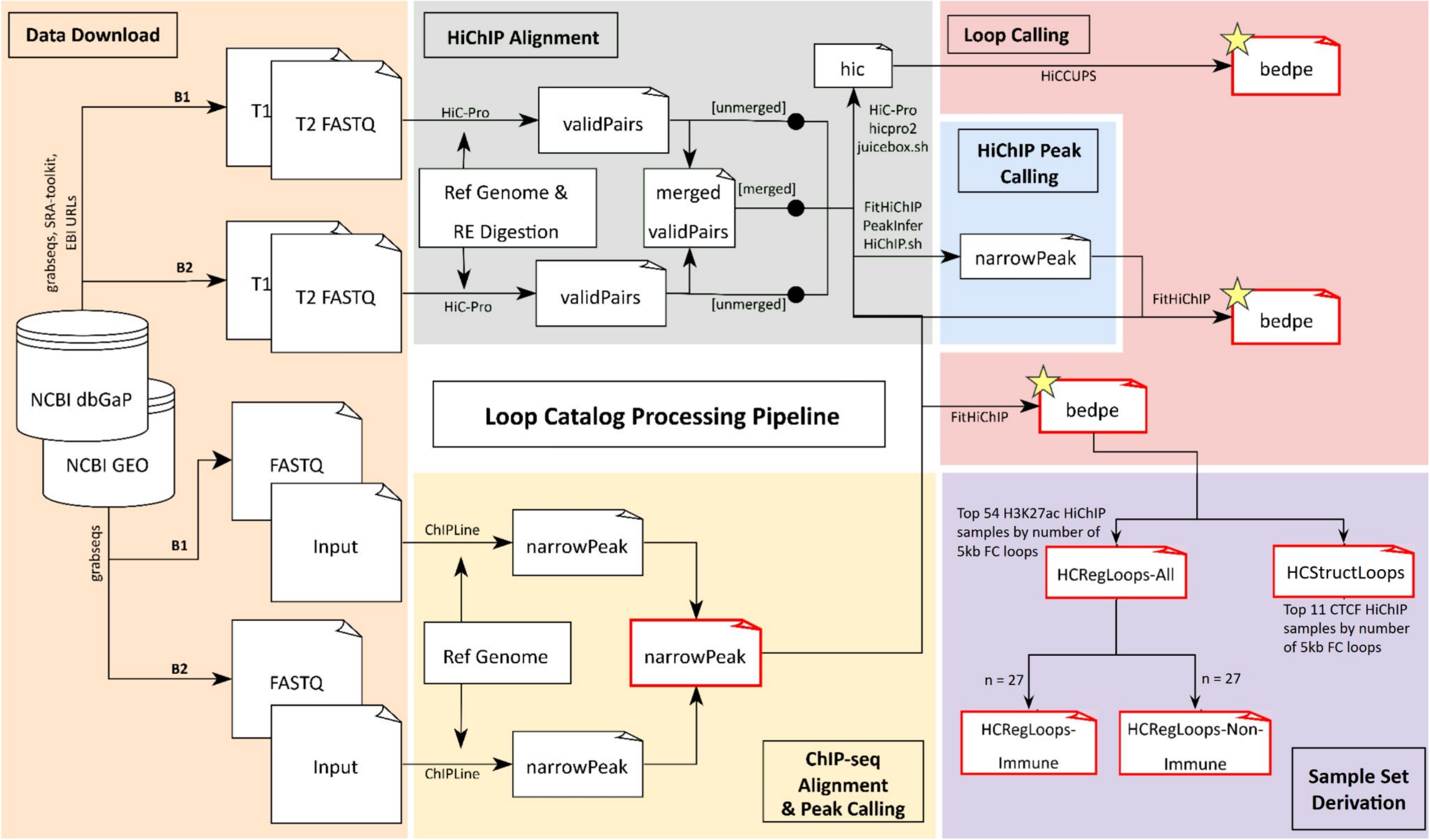
Data Download
grabseqs, SRA-toolkit, EBI URLs
B1
B2
T1
T2 FASTQ
T1
T2 FASTQ
NCBI dbGaP
NCBI GEO
grabseqs
B1
B2
FASTQ
Input
FASTQ
Input
HiChIP Alignment
HiC-Pro
HiC-Pro
validPairs
validPairs
Ref Genome &
RE Digestion
merged
validPairs
[unmerged]
[merged]
[unmerged]
hic
HiC-Pro
hicpro2
juicebox.sh
FitHiChIP
PeakInfer
HiChIP.sh
Loop Calling
HiCCUPS
bedpe
HiChIP Peak
Calling
narrowPeak
FitHiChIP
bedpe
FitHiChIP
bedpe
Loop Catalog Processing Pipeline
ChIPLine
narrowPeak
Ref Genome
narrowPeak
ChIPLine
narrowPeak
ChIP-seq
Alignment
& Peak Calling
Top 54 H3K27ac HiChIP
samples by number of
5kb FC loops
HCRegLoops-All
HCStructLoops
Top 11 CTCF HiChIP
samples by number
of 5kb FC loops
n = 27
n = 27
HCRegLoops-
Immune
HCRegLoops-Non-
Immune
Sample Set
Derivation

Fig. S1: Schematic of the HiChIP and ChIP-seq data processing pipeline. Raw sequencing reads are downloaded from NCBI GEO and dbGaP and are aligned to the reference genome (hg38 or mm10). Loops are called for HiChIP (both unmerged and merged biological replicates, as indicated by the shaded circles) using HiCCUPS and FitHiChIP at the 5kb, 10kb, and 25kb resolutions. Peaks derived from both ChIP-seq and HiChIP are used for FitHiChIP loop calling. High-confidence sample sets of top H3K27ac and CTCF HiChIP samples are curated from the final set of loop calls. Red borders indicate usage in downstream database application analyses. Yellow stars indicate that the data type is available for download from the Loop Catalog web platform.

Fig. S2

A Peak Count and Size (HiChIP-Peaks, FitHiChIP, ChIPLine)

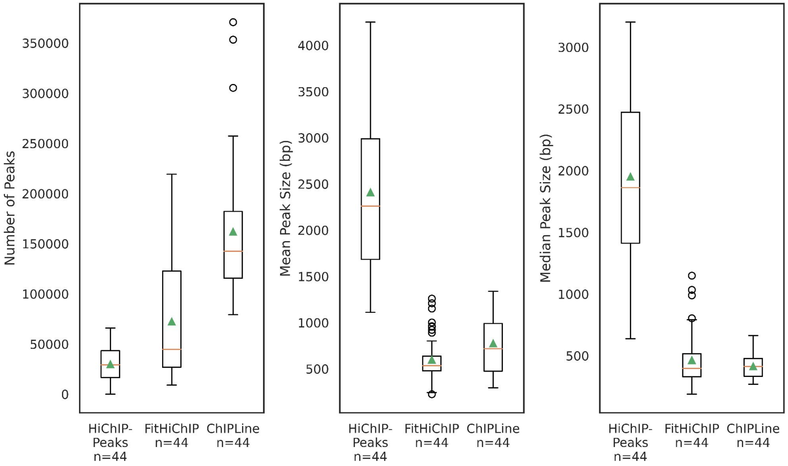

B % Recall of ChIP-seq Peaks Per Total Peak Span

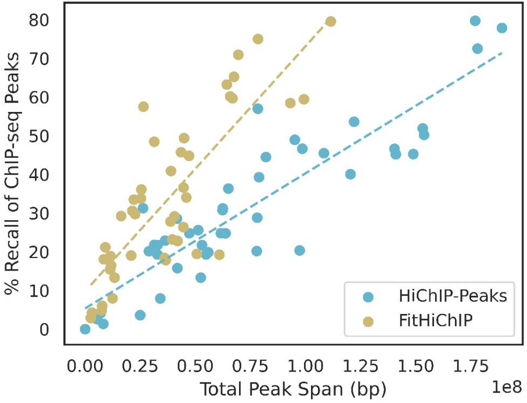

Fig. S2: Comparison of the FitHiChIP (HiChIP-derived), HiChIP-Peaks (HiChIP-derived), and ChIPLine (ChIP-seq) peak calling methods. A) Distributions of number of peaks calls, mean peak size (bp) and median peak size (bp) for 44 human HiChIP and ChIP-seq samples from diverse cell types and protein pulldowns. **B)** scatter plot of recall rate of ChIP-seq peaks by HiChIP-derived peaks versus the total peak span of HiChIP-derived peaks for peaks called by FitHiChIP (green) or HiChIP-Peaks (blue) from HiChIP data.

Fig. S3

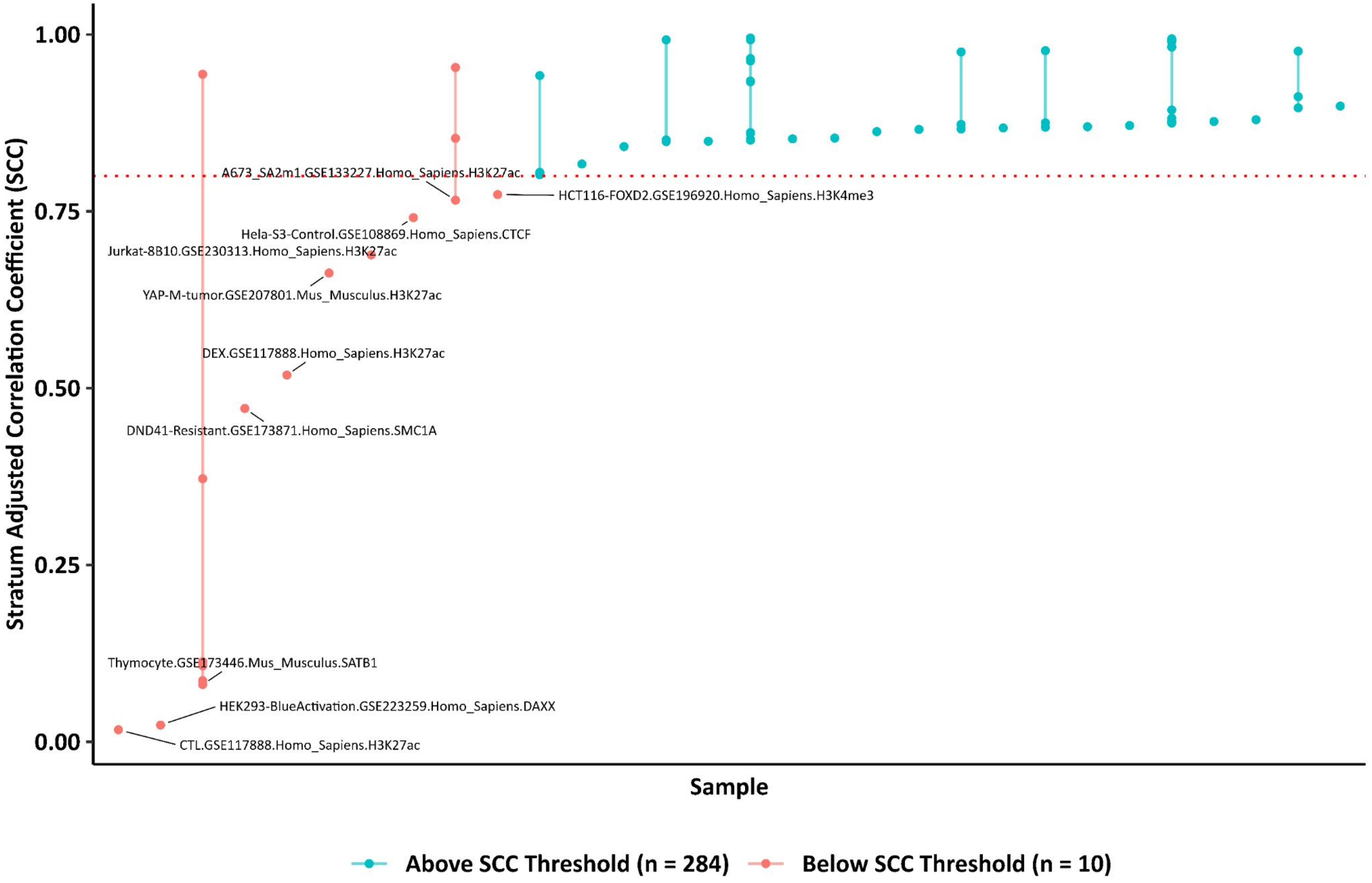

1.00
0.75
0.50
0.25
0.00
Stratum Adjusted Correlation Coefficient (SCC)
Sample
A673_SA2m1.GSE133227.Homo_Sapiens.H3K27ac
HCT116-FOXD2.GSE196920.Homo_Sapiens.H3K4me3
Hela-S3-Control.GSE108869.Homo_Sapiens.CTCF
Jurkat-8B10.GSE230313.Homo_Sapiens.H3K27ac
YAP-M-tumor.GSE207801.Mus_Musculus.H3K27ac
DEX.GSE117888.Homo_Sapiens.H3K27ac
DND41-Resistant.GSE173871.Homo_Sapiens.SMC1A
Thymocyte.GSE173446.Mus_Musculus.SATB1
HEK293-BlueActivation.GSE223259.Homo_Sapiens.DAXX
CTL.GSE117888.Homo_Sapiens.H3K27ac
Above SCC Threshold (n = 284)
Below SCC Threshold (n = 10)

Fig. S3: Reproducibility analysis for HiChIP biological replicates. Stratum adjusted correlation coefficients (SCCs) for all pairwise combinations of HiChIP biological replicates for 294 HiChIP samples with at least two biological replicates were generated using hicreppy. SCCs for pairwise combinations of HiChIP biological replicates are displayed for the 30 HiChIP samples with a minimum SCC below 0.90. SCCs for pairwise combinations of replicates for samples with more than 2 replicates are connected with vertical lines. Samples passed the SCC threshold (0.80, red dashed line) if the SCC of all pairwise combinations of replicates was greater than 0.80 (n = 284 samples, a subset of 20 passing samples shown in blue). Samples which possessed at least one replicate combination with a SCC less than 0.80 did not pass the threshold (n = 10 samples, all shown in red).

Fig. S4

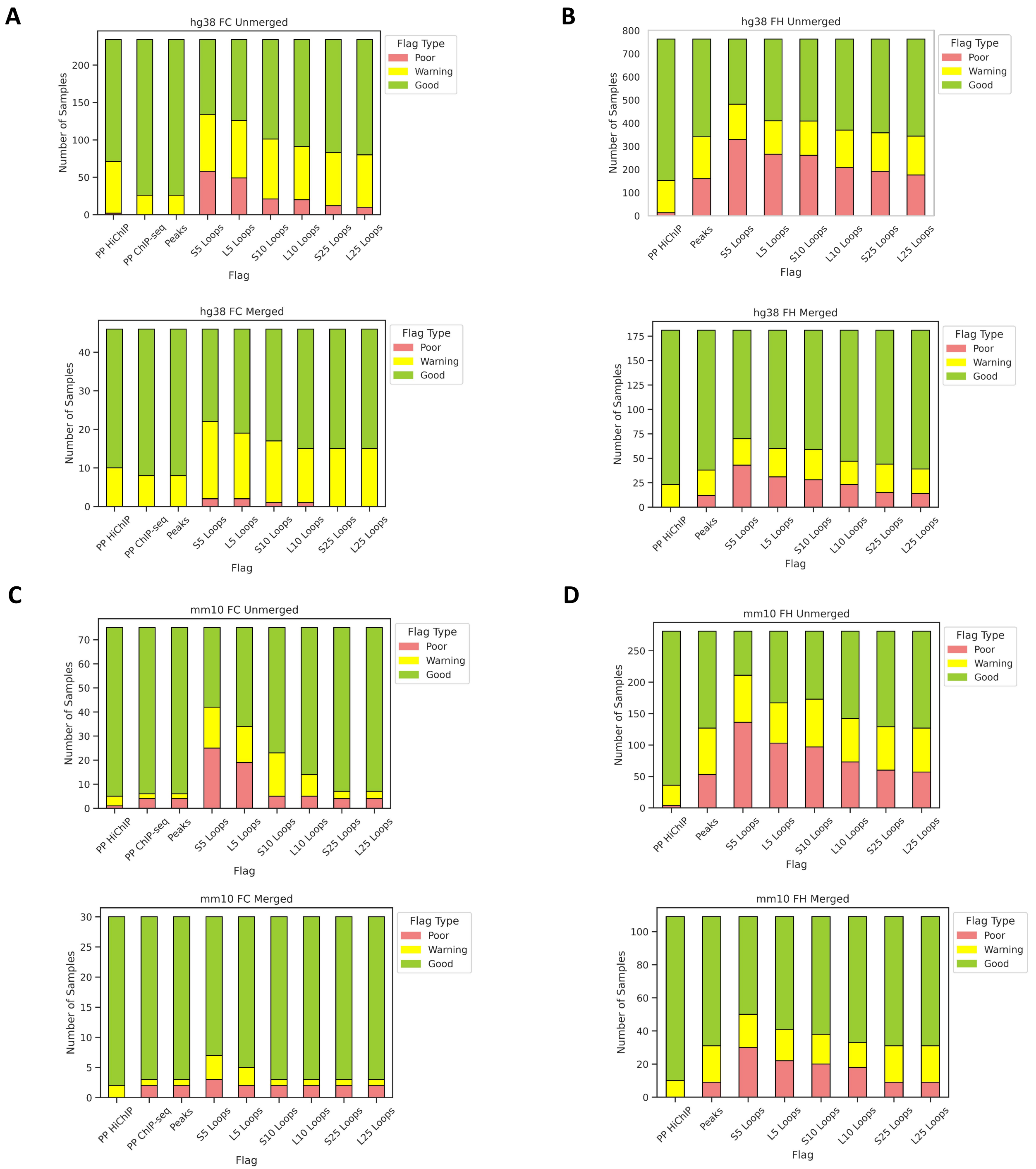
A
hg38 FC Unmerged
Number of Samples
Flag
PP HiChIP
PP ChIP-seq
Peaks
S5 Loops
L5 Loops
S10 Loops
L10 Loops
S25 Loops
L25 Loops
Flag Type
Poor
Warning
Good
B
hg38 FH Unmerged
hg38 FC Merged
hg38 FH Merged
C
mm10 FC Unmerged
D
mm10 FH Unmerged
mm10 FC Merged
mm10 FH Merged

Fig. S4: Assignment of QC Flags to HiChIP Samples. Flags were assigned to all samples at the pre-processing (HiChIP and/or ChIP-seq), peak calling, and loop calling stages according to the system specified in Additional file 10: Table S9. QC flags for FC loops consider loop quality as well as HiChIP pre-processing (PP HiChIP), ChIP-seq pre-processing (PP ChIP-seq), and ChIP-seq peak quality. Flags for FH loops are determined from loop quality, HiChIP pre-processing (HiChIP PP), and peaks inferred from HiChIP. The distribution of each QC flag type is displayed for **A)** human FC loops, unmerged and merged, **B)** human FH loops, unmerged and merged, **C)** mouse FC loops, unmerged and merged, and **D)** mouse FH loops, unmerged and merged.

Fig. S5

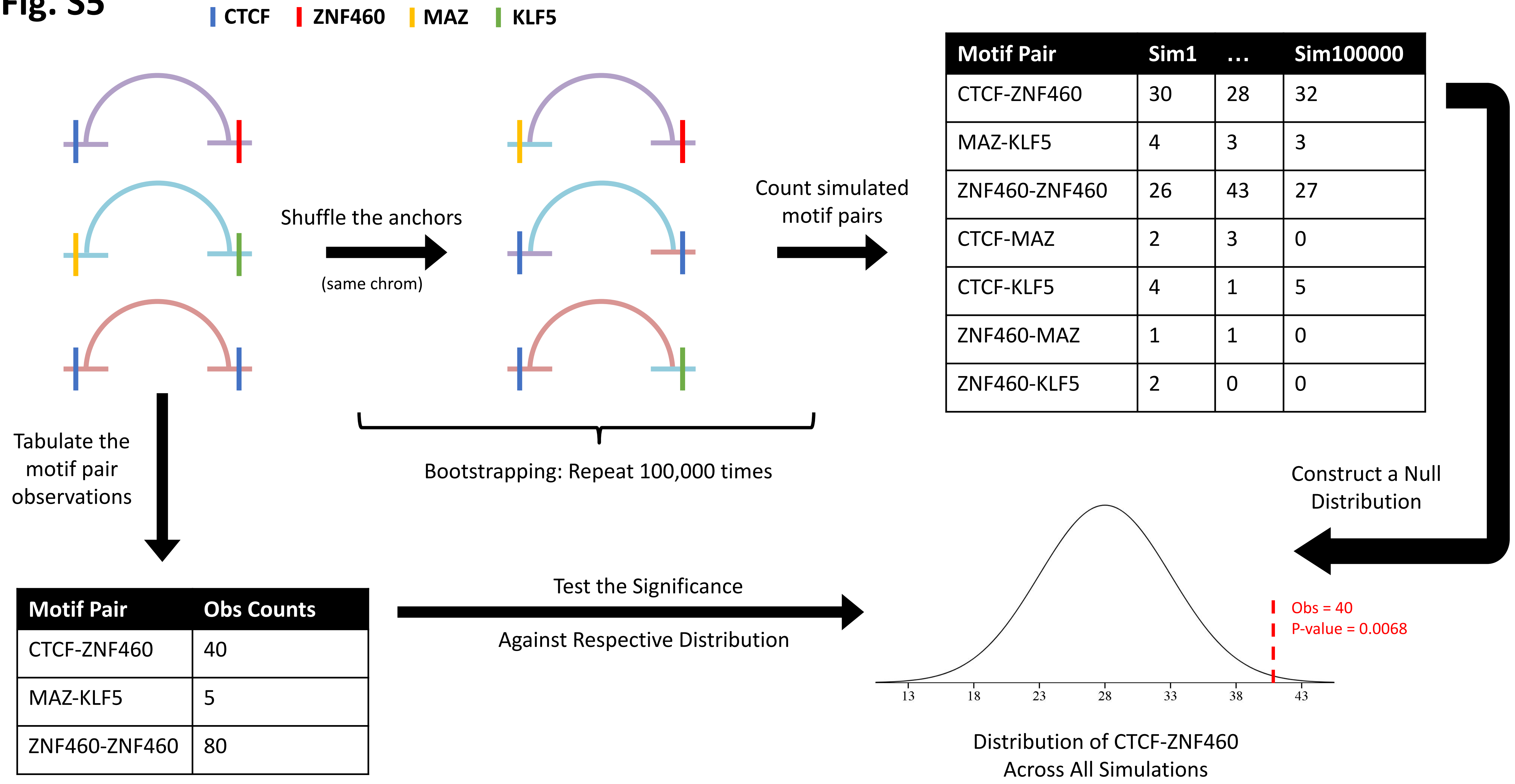

Motif Pair	Sim1	…	Sim100000
CTCF-ZNF460	30	28	32
MAZ-KLF5	4	3	3
ZNF460-ZNF460	26	43	27
CTCF-MAZ	2	3	0
CTCF-KLF5	4	1	5
ZNF460-MAZ	1	1	0
ZNF460-KLF5	2	0	0

Motif Pair	Obs Counts
CTCF-ZNF460	40
MAZ-KLF5	5
ZNF460-ZNF460	80

Fig. S5: Schema of Bootstrap Analysis for Motif Pairs. Loops are depicted with an arc and horizontal lines are used to denote their anchors. Each loop has a distinct color to emphasize the true loop composition and overlapping motifs are depicted with horizontal lines and a separate color for each (top right). Observed motif pairs are tabulated (bottom left). To simulate a new set of loops, anchors are shuffled which brings together new combinations of motifs (top middle). Motif pairs from these simulations are tabulated across 100000 simulations (top right) to build a null distribution (bottom right). The observed counts for a given motif are then evaluated against their respective null distribution (bottom).

Fig. S6

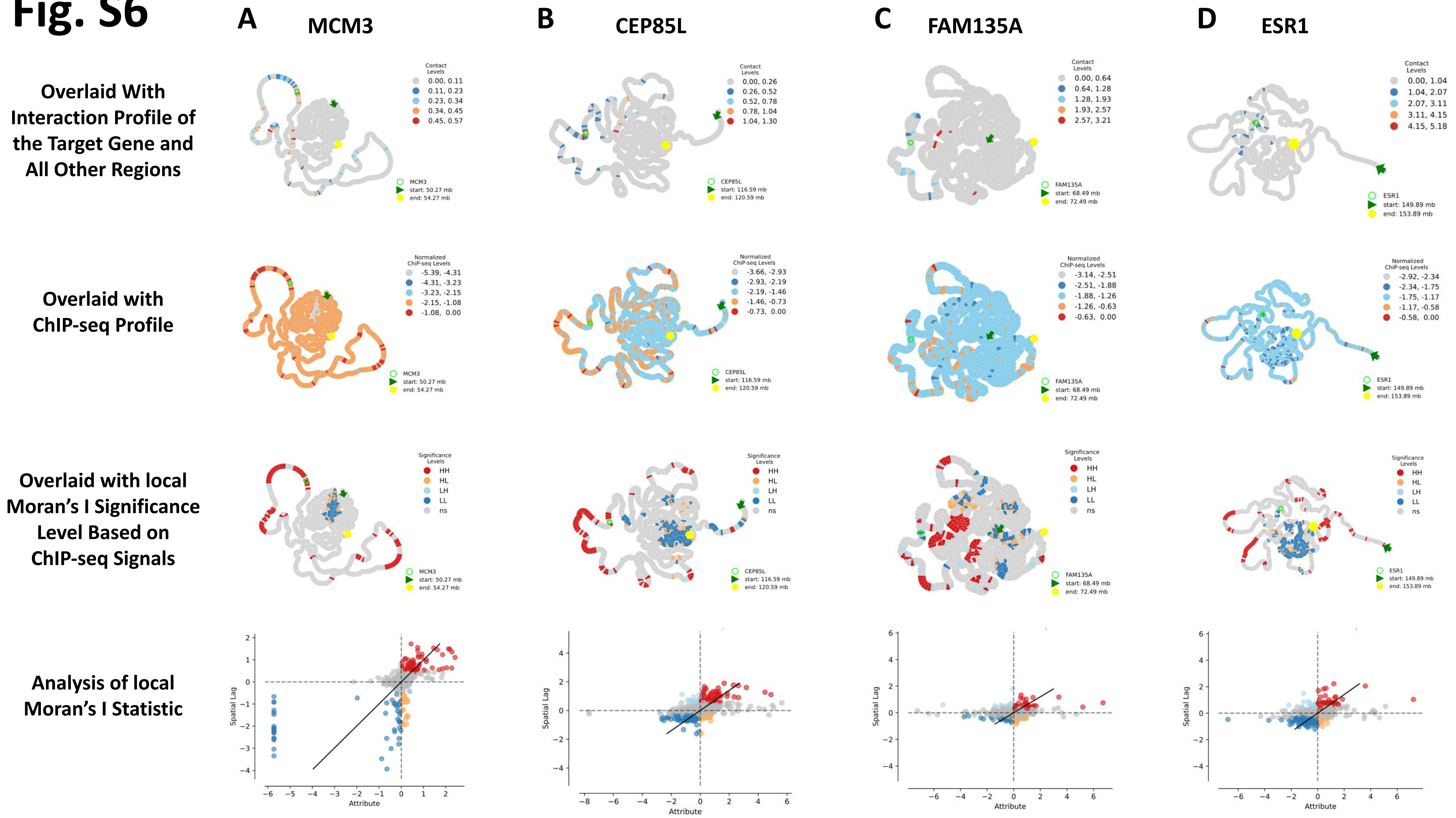
A
MCM3
B
CEP85L
C
FAM135A
D
ESR1
Overlaid With Interaction Profile of the Target Gene and All Other Regions
Overlaid with ChIP-seq Profile
Overlaid with local Moran's I Significance Level Based on ChIP-seq Signals
Analysis of local Moran's I Statistic
Contact Levels
0.00, 0.11
0.11, 0.23
0.23, 0.34
0.34, 0.45
0.45, 0.57
MCM3
start: 50.27 mb
end: 54.27 mb
0.00, 0.26
0.26, 0.52
0.52, 0.78
0.78, 1.04
1.04, 1.30
CEP85L
start: 116.59 mb
end: 120.59 mb
0.00, 0.64
0.64, 1.28
1.28, 1.93
1.93, 2.57
2.57, 3.21
FAM135A
start: 68.49 mb
end: 72.49 mb
0.00, 1.04
1.04, 2.07
2.07, 3.11
3.11, 4.15
4.15, 5.18
ESR1
start: 149.89 mb
end: 153.89 mb
Normalized ChiP-seq Levels
-5.39, -4.31
-4.31, -3.23
-3.23, -2.15
-2.15, -1.08
-1.08, 0.00
-3.66, -2.93
-2.93, -2.19
-2.19, -1.46
-1.46, -0.73
-0.73, 0.00
-3.14, -2.51
-2.51, -1.88
-1.88, -1.26
-1.26, -0.63
-0.63, 0.00
-2.92, -2.34
-2.34, -1.75
-1.75, -1.17
-1.17, -0.58
-0.58, 0.00
Significance Levels
HH
HL
LH
LL
ns
Spatial Lag
Attribute

Fig. S6: Visualization of chromatin conformation and regulatory elements surrounding genes using 2D embedding models. A) Visualization of chromatin conformation as 2D embedding models for MCM3 (visualized as a green circle). From top to bottom, the first three subpanels contain a visualization of the 2D model with bins colored based on the total number of contacts within this 4mb region (first), the level of ChIP-seq signals (second), and the local Moran's I statistical significance value (third). The last sub panel contains a scatter plot of local Moran's I displaying attribute (ChIP-seq signal) versus spatial lag. **B-D)** similar results for genes CEP85L, FAM135A, and ESR1, respectively.

Fig. S7

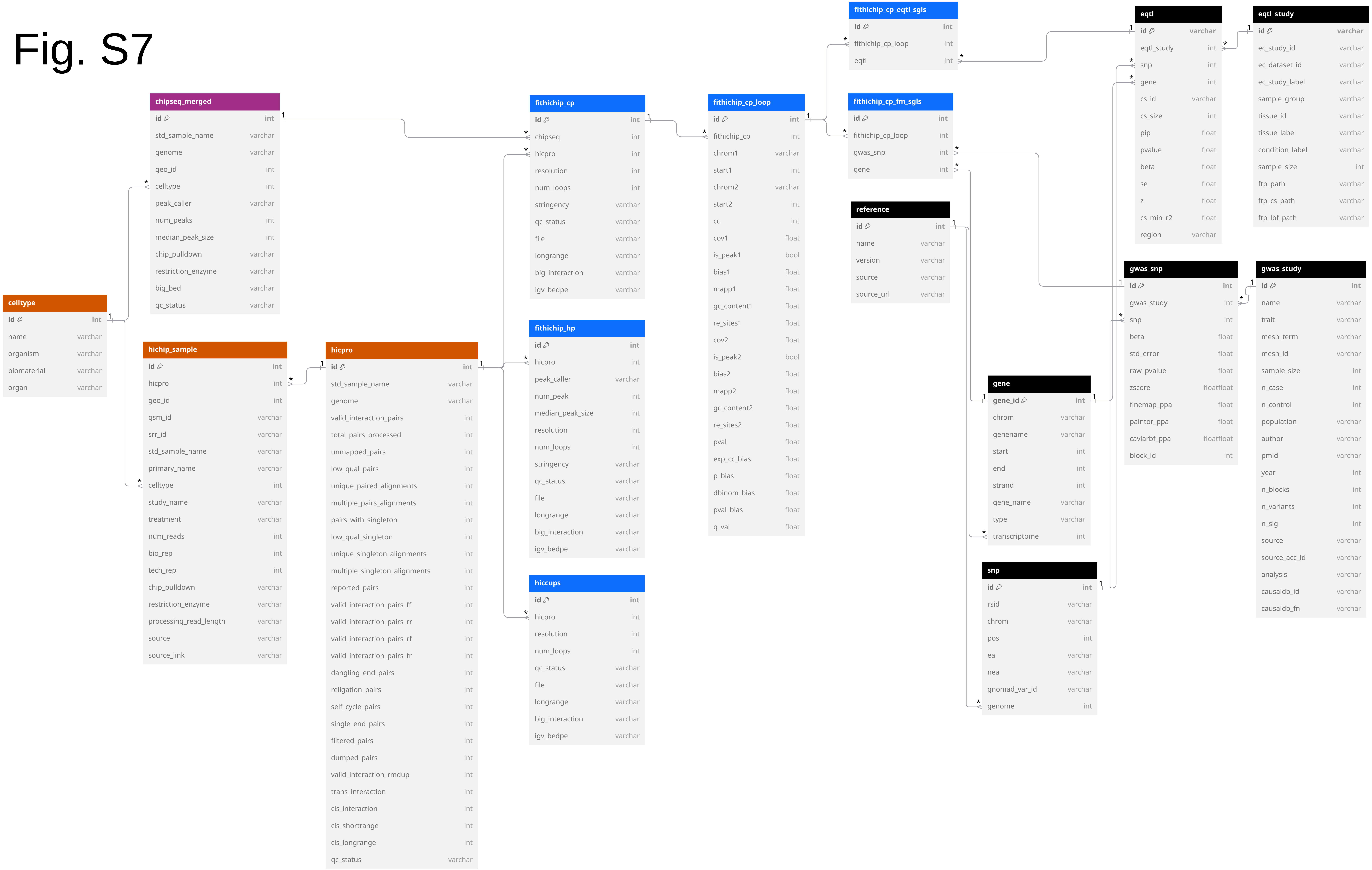

chipseq_merged
id int
std_sample_name varchar
genome varchar
geo_id int
celltype int
peak_caller varchar
num_peaks int
median_peak_size int
chip_pulldown varchar
restriction_enzyme varchar
big_bed varchar
qc_status varchar
celltype
id int
name varchar
organism varchar
biomaterial varchar
organ varchar
hichip_sample
id int
hicpro int
geo_id int
gsm_id varchar
srr_id varchar
std_sample_name varchar
primary_name varchar
celltype int
study_name varchar
treatment varchar
num_reads int
bio_rep int
tech_rep int
chip_pulldown varchar
restriction_enzyme varchar
processing_read_length varchar
source varchar
source_link varchar
hicpro
id int
std_sample_name varchar
genome varchar
valid_interaction_pairs int
total_pairs_processed int
unmapped_pairs int
low_qual_pairs int
unique_paired_alignments int
multiple_pairs_alignments int
pairs_with_singleton int
low_qual_singleton int
unique_singleton_alignments int
multiple_singleton_alignments int
reported_pairs int
valid_interaction_pairs_ff int
valid_interaction_pairs_rr int
valid_interaction_pairs_rf int
valid_interaction_pairs_fr int
dangling_end_pairs int
religation_pairs int
self_cycle_pairs int
single_end_pairs int
filtered_pairs int
dumped_pairs int
valid_interaction_rmdup int
trans_interaction int
cis_interaction int
cis_shortrange int
cis_longrange int
qc_status varchar
fithichip_cp
id int
chipseq int
hicpro int
resolution int
num_loops int
stringency varchar
qc_status varchar
file varchar
longrange varchar
big_interaction varchar
igv_bedpe varchar
fithichip_hp
id int
hicpro int
peak_caller varchar
num_peak int
median_peak_size int
resolution int
num_loops int
stringency varchar
qc_status varchar
file varchar
longrange varchar
big_interaction varchar
igv_bedpe varchar
hiccups
id int
hicpro int
resolution int
num_loops int
qc_status varchar
file varchar
longrange varchar
big_interaction varchar
igv_bedpe varchar
fithichip_cp_loop
id int
fithichip_cp int
chrom1 varchar
start1 int
chrom2 varchar
start2 int
cc int
cov1 float
is_peak1 bool
bias1 float
mapp1 float
gc_content1 float
re_sites1 float
cov2 float
is_peak2 bool
bias2 float
mapp2 float
gc_content2 float
re_sites2 float
pval float
exp_cc_bias float
p_bias float
dbinom_bias float
pval_bias float
q_val float
fithichip_cp_eqtl_sgls
id int
fithichip_cp_loop int
eqtl int
fithichip_cp_fm_sgls
id int
fithichip_cp_loop int
gwas_snp int
gene int
reference
id int
name varchar
version varchar
source varchar
source_url varchar
gene
gene_id int
chrom varchar
genename varchar
start int
end int
strand int
gene_name varchar
type varchar
transcriptome int
snp
id int
rsid varchar
chrom varchar
pos int
ea varchar
nea varchar
gnomad_var_id varchar
genome int
eqtl
id varchar
eqtl_study int
snp int
gene int
cs_id varchar
cs_size int
pip float
pvalue float
beta float
se float
z float
cs_min_r2 float
region varchar
eqtl_study
id varchar
ec_study_id varchar
ec_dataset_id varchar
ec_study_label varchar
sample_group varchar
tissue_id varchar
tissue_label varchar
condition_label varchar
sample_size int
ftp_path varchar
ftp_cs_path varchar
ftp_lbf_path varchar
gwas_snp
id int
gwas_study int
snp int
beta float
std_error float
raw_pvalue float
zscore floatfloat
finemap_ppa float
paintor_ppa float
caviarbf_ppa floatfloat
block_id int
gwas_study
id int
name varchar
trait varchar
mesh_term varchar
mesh_id varchar
sample_size int
n_case int
n_control int
population varchar
author varchar
pmid varchar
year int
n_blocks int
n_variants int
n_sig int
source varchar
source_acc_id varchar
analysis varchar
causaldb_id varchar
causaldb_fn varchar

Fig. S7: Schema of the Loop Catalog Database. Tables with orange contain metadata for each HiChIP sample, purple indicates ChIP-seq data, blue indicates tables with loop or loop-associated data, and black indicates extra reference datasets (i.e. gene coordinates from GENCODE).

Fig. S8

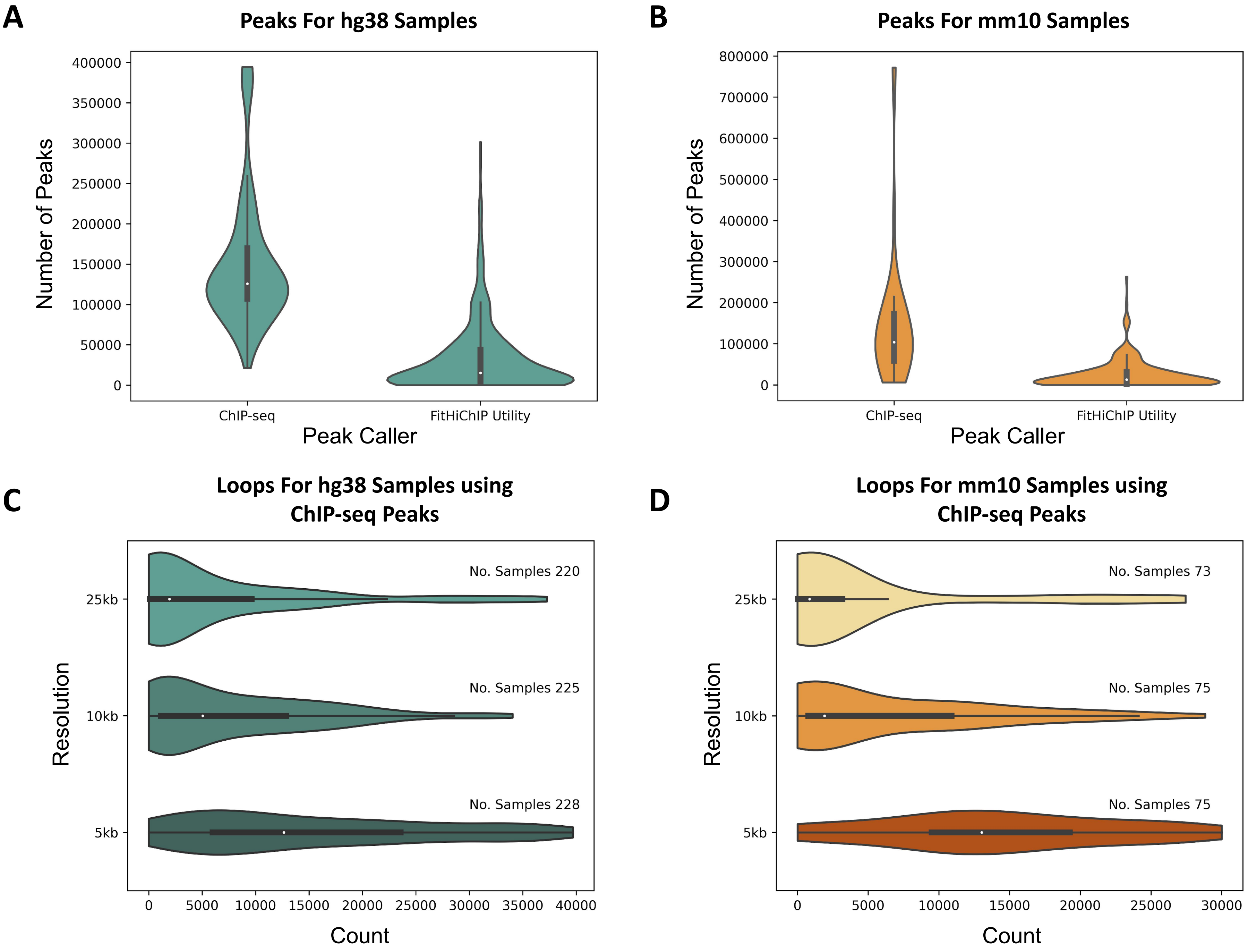
A
Peaks For hg38 Samples
Number of Peaks
400000
350000
300000
250000
200000
150000
100000
50000
0
ChIP-seq
FitHiChIP Utility
Peak Caller
B
Peaks For mm10 Samples
Number of Peaks
800000
700000
600000
500000
400000
300000
200000
100000
0
ChIP-seq
FitHiChIP Utility
Peak Caller
C
Loops For hg38 Samples using ChIP-seq Peaks
Resolution
25kb
10kb
5kb
No. Samples 220
No. Samples 225
No. Samples 228
0
5000
10000
15000
20000
25000
30000
35000
40000
Count
D
Loops For mm10 Samples using ChIP-seq Peaks
Resolution
25kb
10kb
5kb
No. Samples 73
No. Samples 75
No. Samples 75
0
5000
10000
15000
20000
25000
30000
Count

Fig. S8: Selected summary of peaks and loops of the Loop Catalog. A and **B)** Number of peaks derived from ChIP-seq data for human and mouse, respectively. **C** and **D)** FitHiChIP loops derived for samples with ChIP-seq data at different resolutions for human and mouse, respectively.

Fig. S9

A Comparison of Alignment Metrics (HiC-Pro vs. distiller-nf)

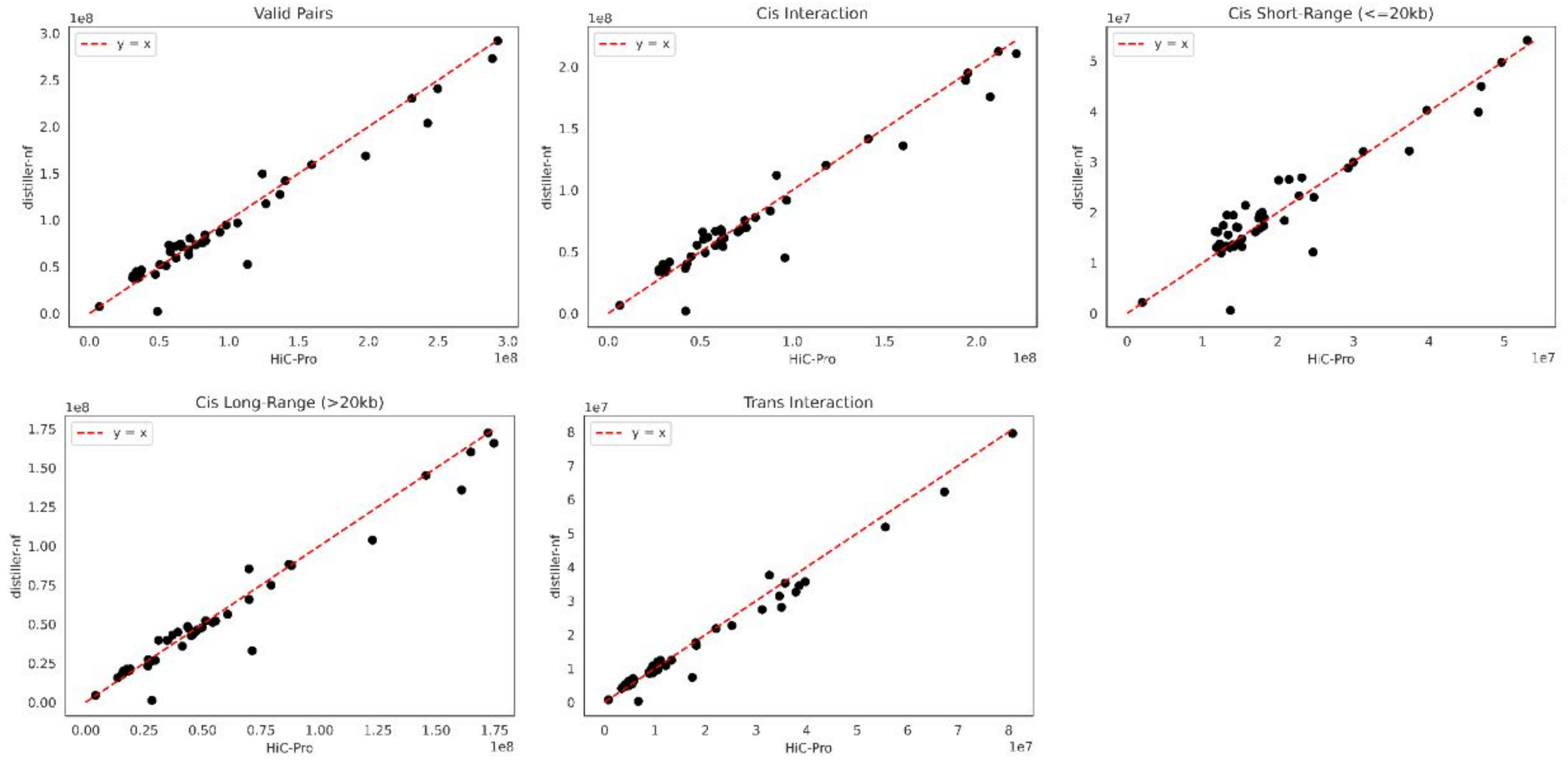

B Comparison of Alignment Metrics (HiC-Pro vs. Juicer)

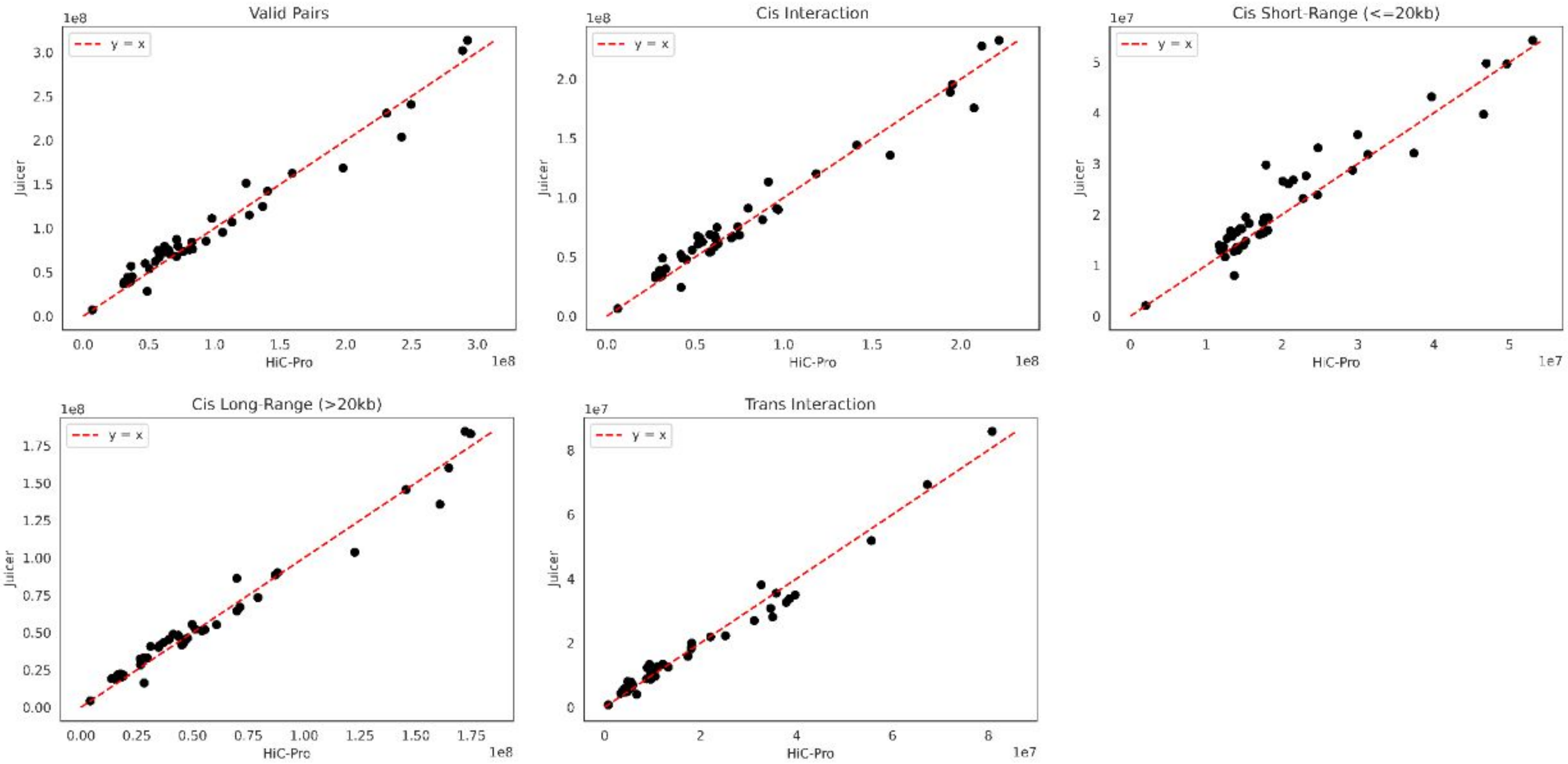

C Loop Overlap (1kb Slack)

CD34+-Cord-Blood.GSE165207.
Homo_Sapiens.H3K27ac.b1

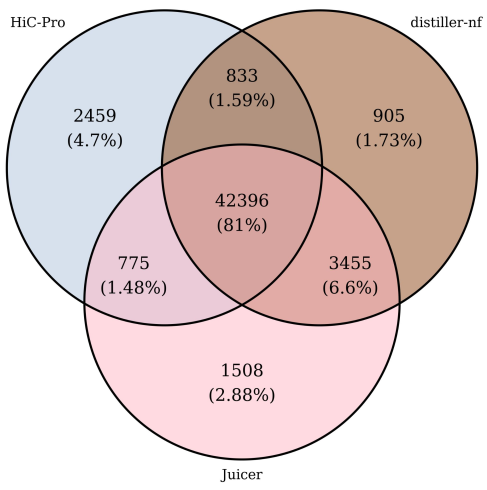

D Loop Strength for Loops Derived from HiC-Pro, distiller-nf, or Juicer

CD34+-Cord-Blood.GSE165207.Homo_Sapiens.H3K27ac.b1

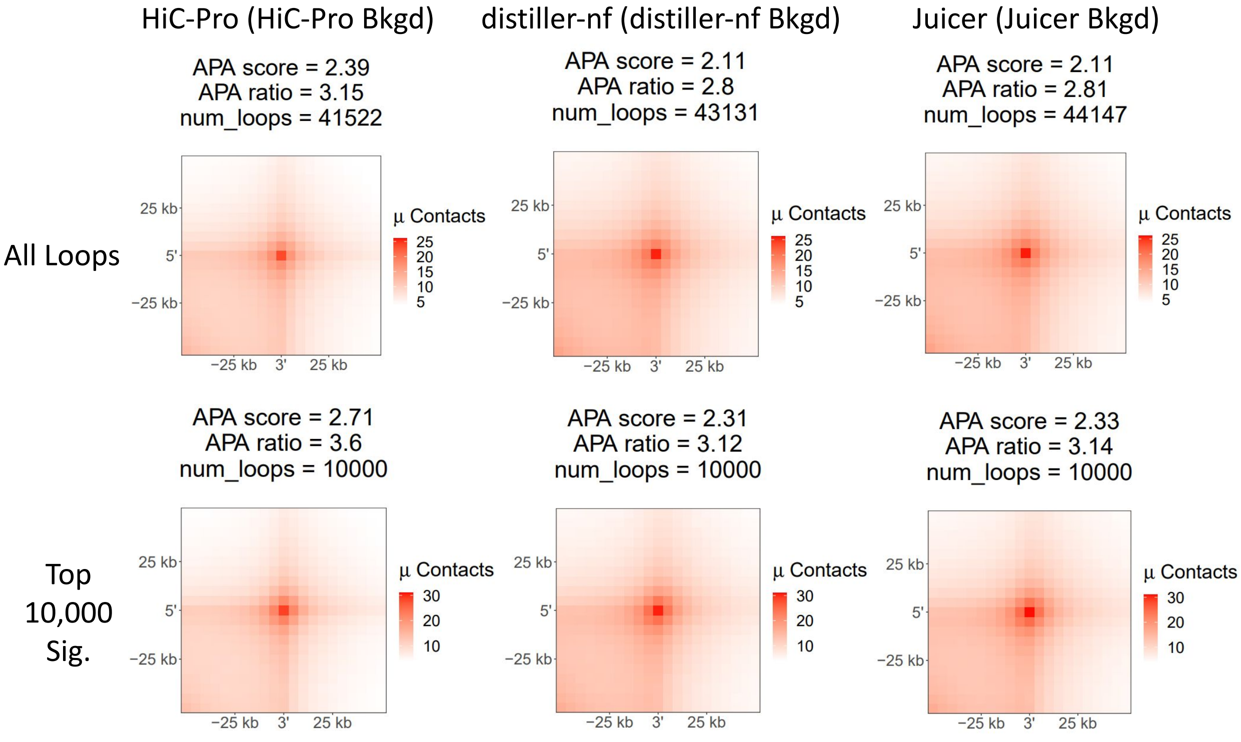

Fig. S9: Comparison of HiC-Pro, distiller-nf, and Juicer for HiChIP read alignment. Scatterplots compare the number of valid pairs, cis interaction pairs, cis short-range pairs (<=20kb), cis long-range pairs (>20kb), and trans interaction pairs for **A)** HiC-Pro vs. distiller-nf and **B)** HiC-Pro vs. Juicer for 45 human HiChIP samples from diverse cell types and protein pulldowns. Distiller-nf UU (unique-unique) and UR (unique-rescue or rescue-unique) pairs were retained for this comparison, and for all tools, pairs with a cis distance less than 1kb were discarded. **C)** Venn diagram displaying loop overlap between loops called from pairs derived using HiC-Pro, distiller-nf, or Juicer for the CD34+-Cord-Blood.GSE165207.Homo_Sapiens.H3K27ac.b1 sample. FitHiChIP loop-calling was performed at the 5kb resolution using the stringent background model. +/- 1 bin slack was allowed. **D)** APA plots for all 5kb loops (top row) or the top 10,000 significant 5kb loops by q-value (bottom row) derived from pairs from each of the three alignment methods (HiC-Pro, distiller-nf, or Juicer) for the CD34+-Cord-Blood.GSE165207.Homo_Sapiens.H3K27ac.b1 sample. APA for each tool was performed using the ICE-balanced contact matrix that was generated by the same tool as background. All loops were called by FitHiChIP using ChIP-seq peaks and the stringent background model.

Fig. S10

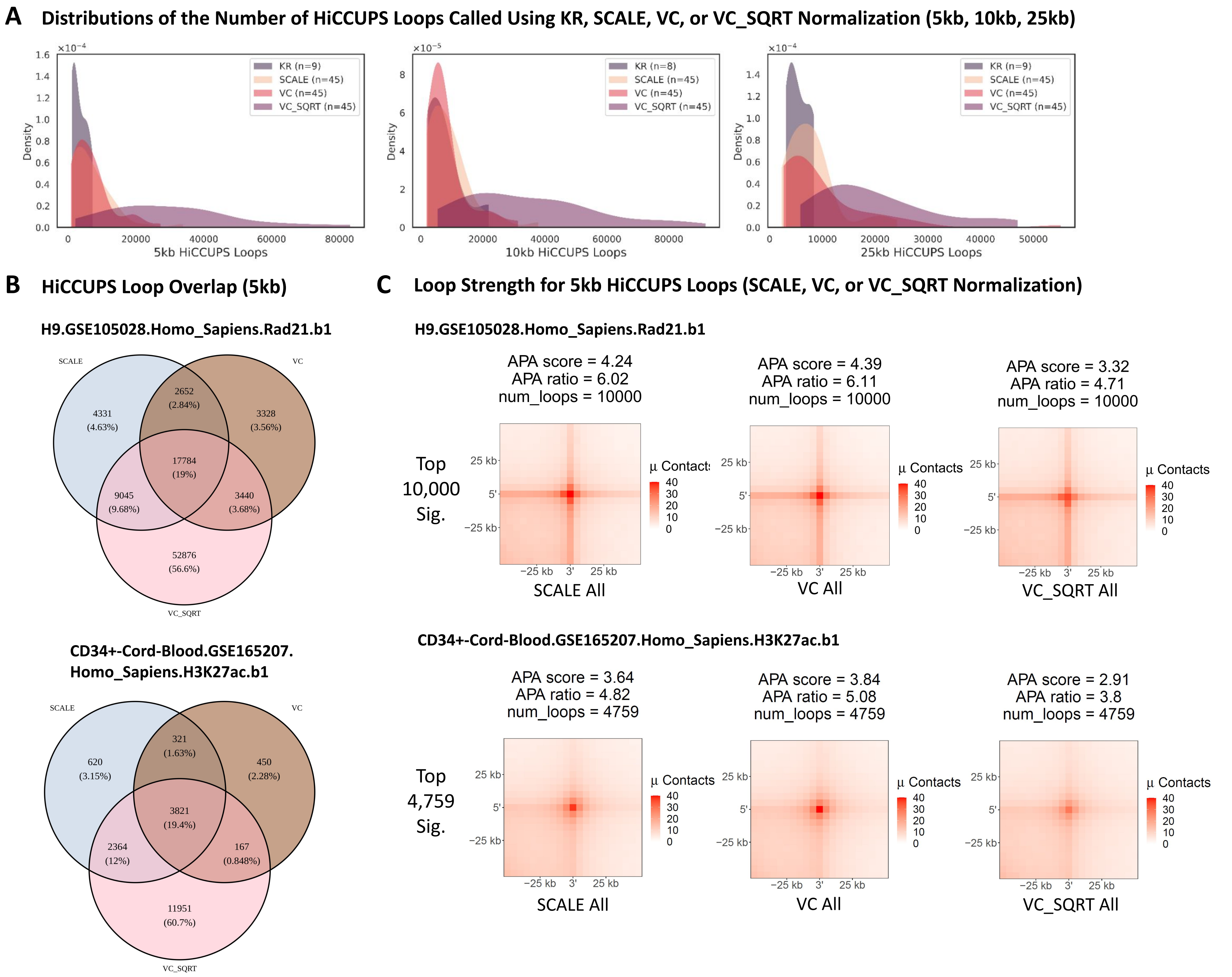
A
Distributions of the Number of HiCCUPS Loops Called Using KR, SCALE, VC, or VC_SQRT Normalization (5kb, 10kb, 25kb)
Density
KR (n=9)
SCALE (n=45)
VC (n=45)
VC_SQRT (n=45)
5kb HiCCUPS Loops
KR (n=8)
SCALE (n=45)
VC (n=45)
VC_SQRT (n=45)
10kb HiCCUPS Loops
KR (n=9)
SCALE (n=45)
VC (n=45)
VC_SQRT (n=45)
25kb HiCCUPS Loops
B
HiCCUPS Loop Overlap (5kb)
H9.GSE105028.Homo_Sapiens.Rad21.b1
SCALE
VC
VC_SQRT
4331 (4.63%)
2652 (2.84%)
3328 (3.56%)
17784 (19%)
9045 (9.68%)
3440 (3.68%)
52876 (56.6%)
CD34+-Cord-Blood.GSE165207.
Homo_Sapiens.H3K27ac.b1
SCALE
VC
VC_SQRT
620 (3.15%)
321 (1.63%)
450 (2.28%)
3821 (19.4%)
2364 (12%)
167 (0.848%)
11951 (60.7%)
C
Loop Strength for 5kb HiCCUPS Loops (SCALE, VC, or VC_SQRT Normalization)
H9.GSE105028.Homo_Sapiens.Rad21.b1
Top 10,000 Sig.
APA score = 4.24
APA ratio = 6.02
num_loops = 10000
SCALE All
APA score = 4.39
APA ratio = 6.11
num_loops = 10000
VC All
APA score = 3.32
APA ratio = 4.71
num_loops = 10000
VC_SQRT All
µ Contacts
CD34+-Cord-Blood.GSE165207.Homo_Sapiens.H3K27ac.b1
Top 4,759 Sig.
APA score = 3.64
APA ratio = 4.82
num_loops = 4759
SCALE All
APA score = 3.84
APA ratio = 5.08
num_loops = 4759
VC All
APA score = 2.91
APA ratio = 3.8
num_loops = 4759
VC_SQRT All
µ Contacts

Fig. S10: Comparison of the Knight-Ruiz (KR), SCALE, Vanilla Coverage (VC), and Vanilla Coverage Square Root (VC_SQRT) matrix normalization methods used for HiCCUPS loop calling. **A)** Distributions of number of HiCCUPS loops called at the 5kb (left), 10kb (center), and 25kb (right) resolutions from HiChIP normalized using either KR (n=8 samples out of the full sample set (n=45) for which KR converged for all chromosomes), SCALE (n=45), VC (n=45), or VC_SQRT (n=45) normalization methods. **B)** For the two samples H9.GSE105028.Homo_Sapiens.Rad21.b1 (top) and CD34+-Cord-Blood.GSE165207.Homo_Sapiens.H3K27ac.b1 (bottom), venn diagrams display the overlap of loops called by HiCCUPS at 5kb resolution using either SCALE, VC, or VC_SQRT normalization methods. No slack was allowed. **C)** For H9.GSE105028.Homo_Sapiens.Rad21.b1 (top) and CD34+-Cord-Blood.GSE165207.Homo_Sapiens.H3K27ac.b1 (bottom), APA plots display loop strength for the top significant loops by the donut FDR called by HiCCUPS using each normalization method. For H9.GSE105028.Homo_Sapiens.Rad21.b1, all samples had over 27,000 loop calls (SCALE All: n = 33,812 loops; VC All: n = 27,204 loops; VC_SQRT: n = 83,145 loops); hence, the top 10,000 significant loops were selected. For CD34+-Cord-Blood.GSE165207.Homo_Sapiens.H3K27ac.b1, VC All had 4,759 total loops (SCALE All: n = 7,126 loops; VC_SQRT: n = 18,303 loops), so 4,759 was taken as the number of top significant loops selected for each method. The ICE-normalized contact matrix generated by HiC-Pro was used as the background for all APA.

Fig. S11

A

Number of Loop Calls by Protein Pulldown (hg38)

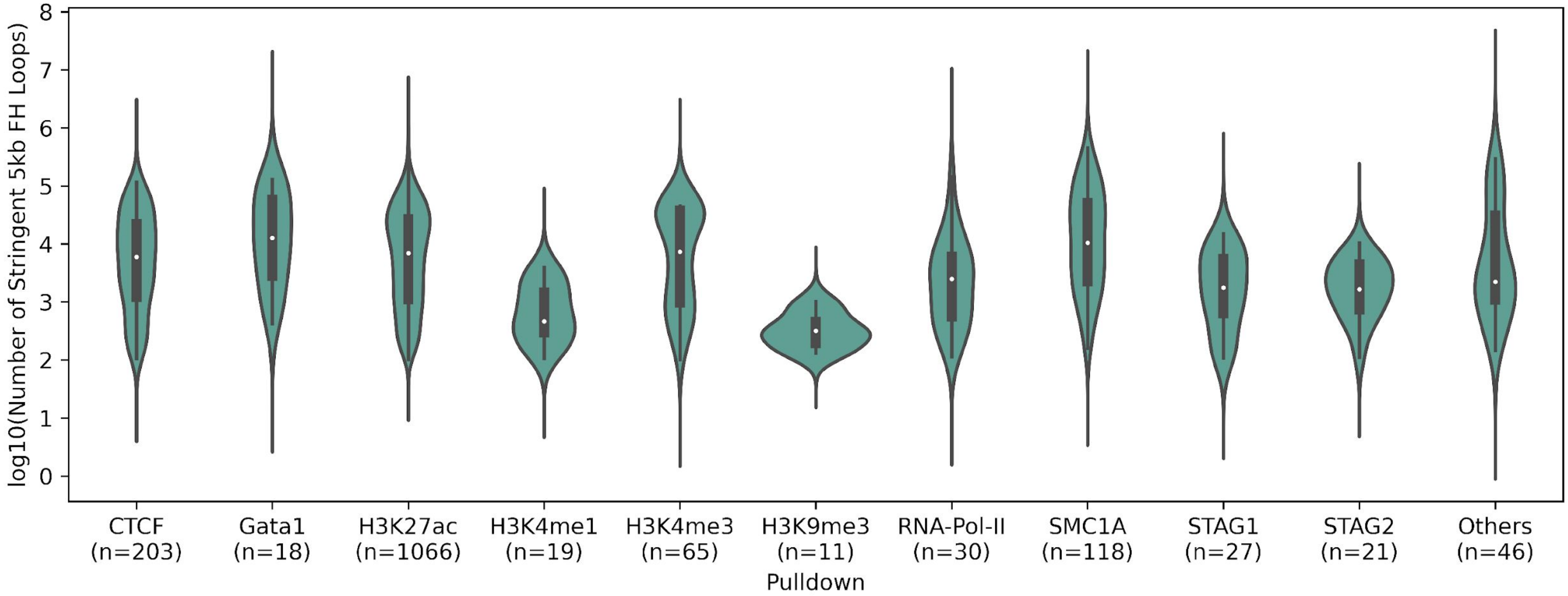

B

Number of Loop Calls by Protein Pulldown (mm10)

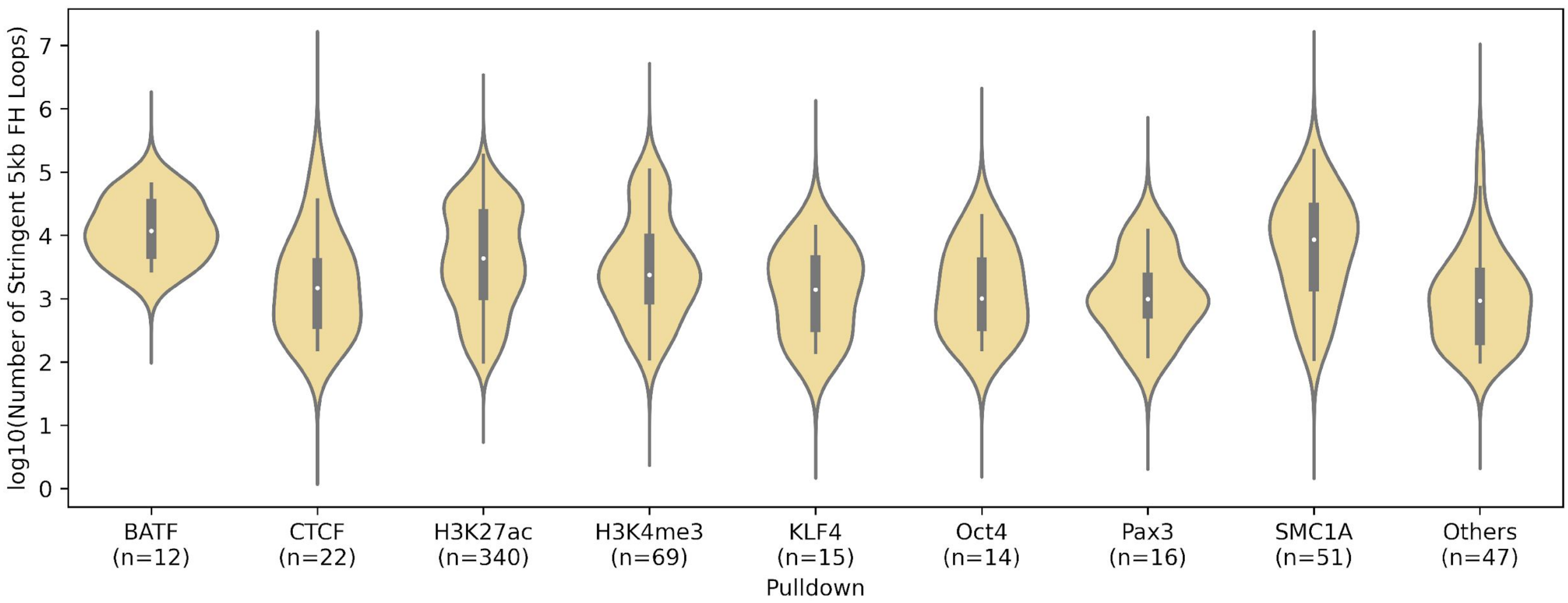

Fig. S11: Number of FH Loop Calls by Protein Pulldown. Distributions of number of FH loop calls by protein pulldown are displayed for **A)** human and **B)** mouse samples with >100 loops. Proteins represented by >10 samples are individually displayed while all others are grouped into the "Other" category. These include proteins such as RNA-Pol-II, GATA1, STAG1, STAG2, RAD21, etc.

Fig. S12

A **Distribution of Loops Sizes for 5kb FC Loops (distance < 1 million bp) (hg38)**

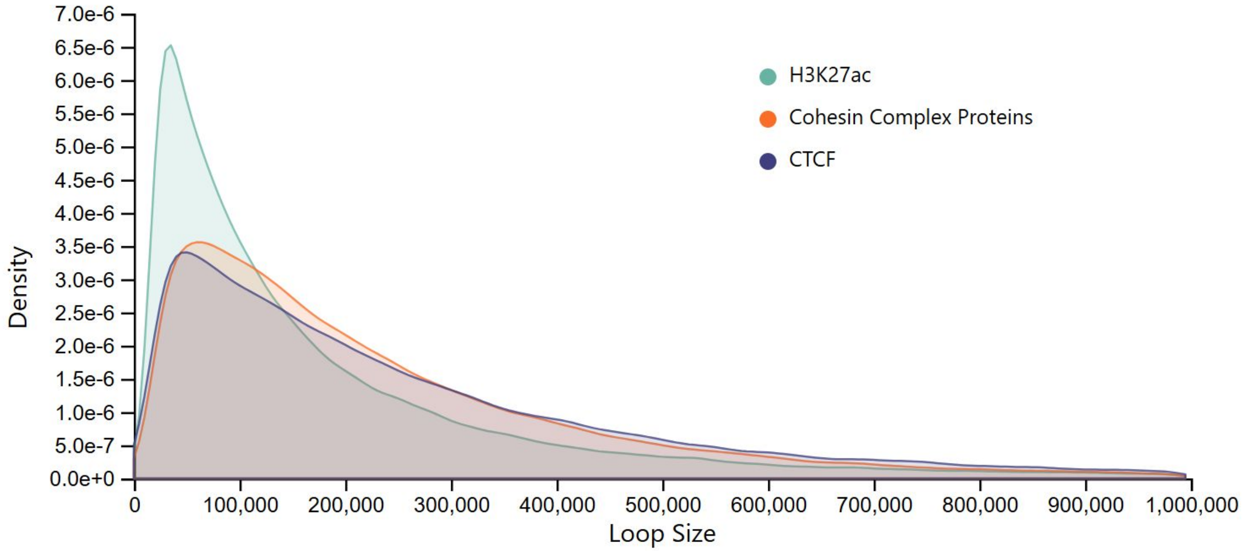

B **Distribution of Loops Sizes for 5kb FC Loops (distance < 1 million bp) (mm10)**

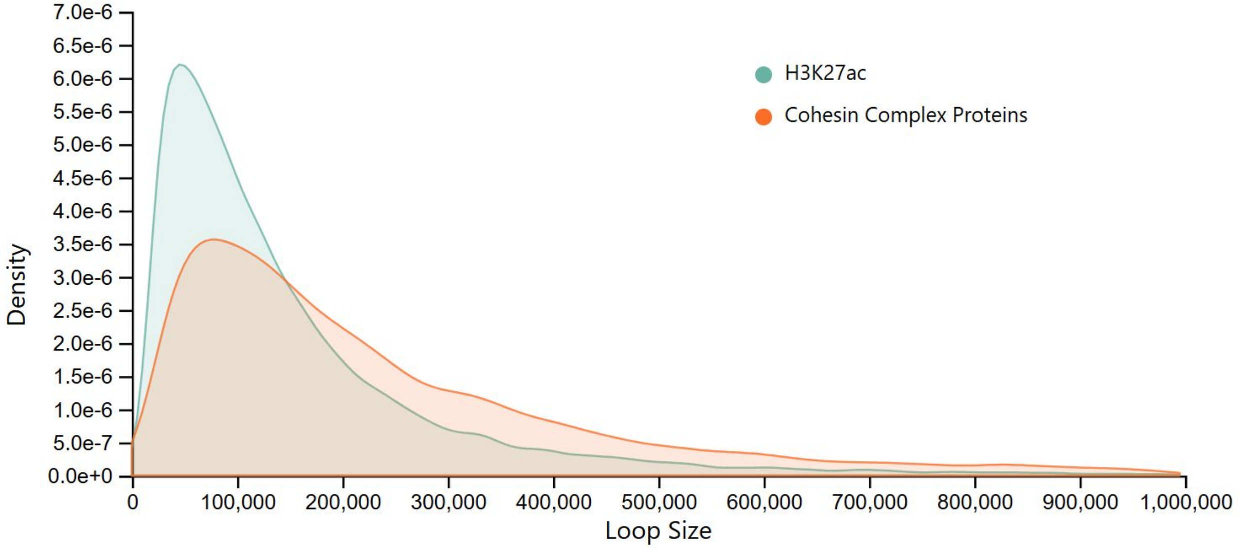

Fig. S12: Loop Size of FC Loops Stratified by Protein Pulldown. Distributions of FC loop sizes for H3K27ac, CTCF and cohesin complex proteins in **A)** human and **B)** mouse samples.

Fig. S13

A

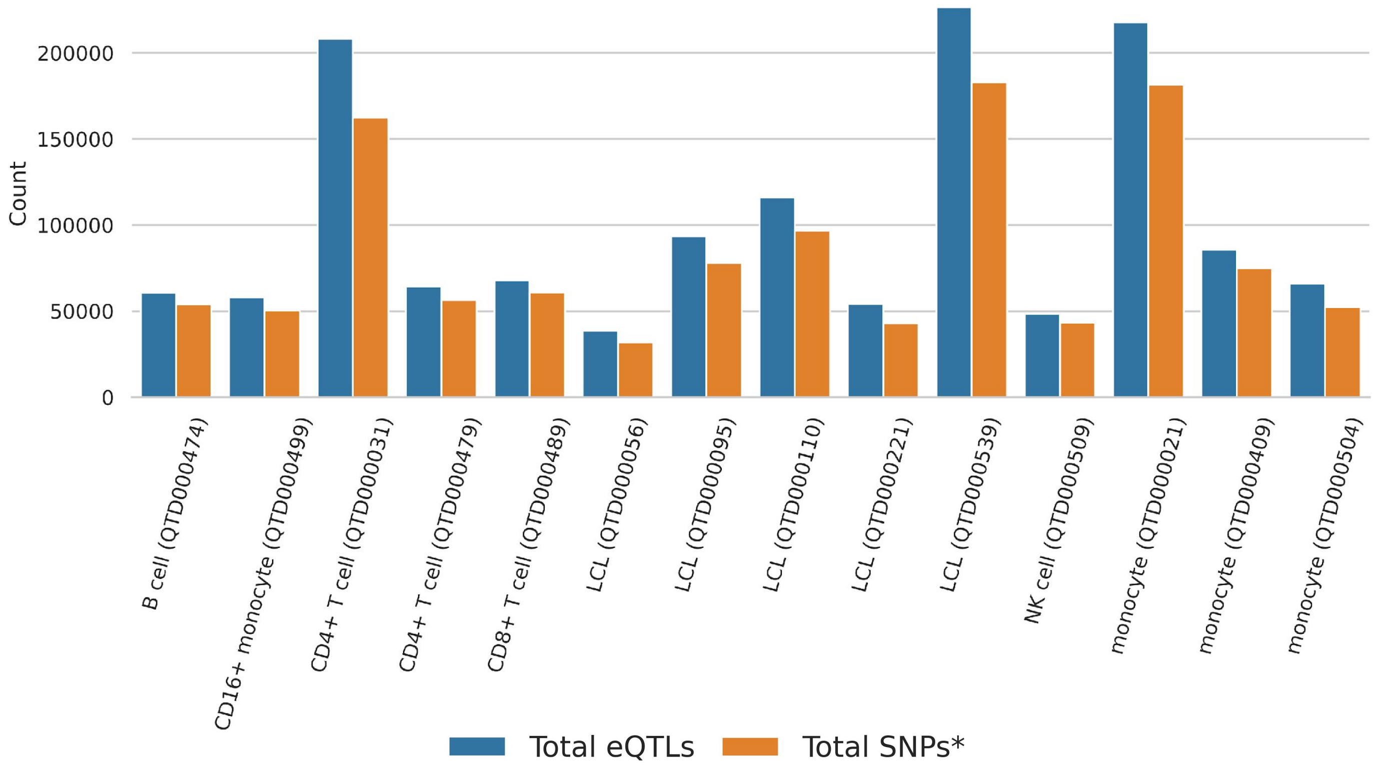

Count
0
50000
100000
150000
200000
B cell (QTD000474)
CD16+ monocyte (QTD000499)
CD4+ T cell (QTD000031)
CD4+ T cell (QTD000479)
CD8+ T cell (QTD000489)
LCL (QTD000056)
LCL (QTD000095)
LCL (QTD000110)
LCL (QTD000221)
LCL (QTD000539)
NK cell (QTD000509)
monocyte (QTD000021)
monocyte (QTD000409)
monocyte (QTD000504)
Total eQTLs
Total SNPs*

B

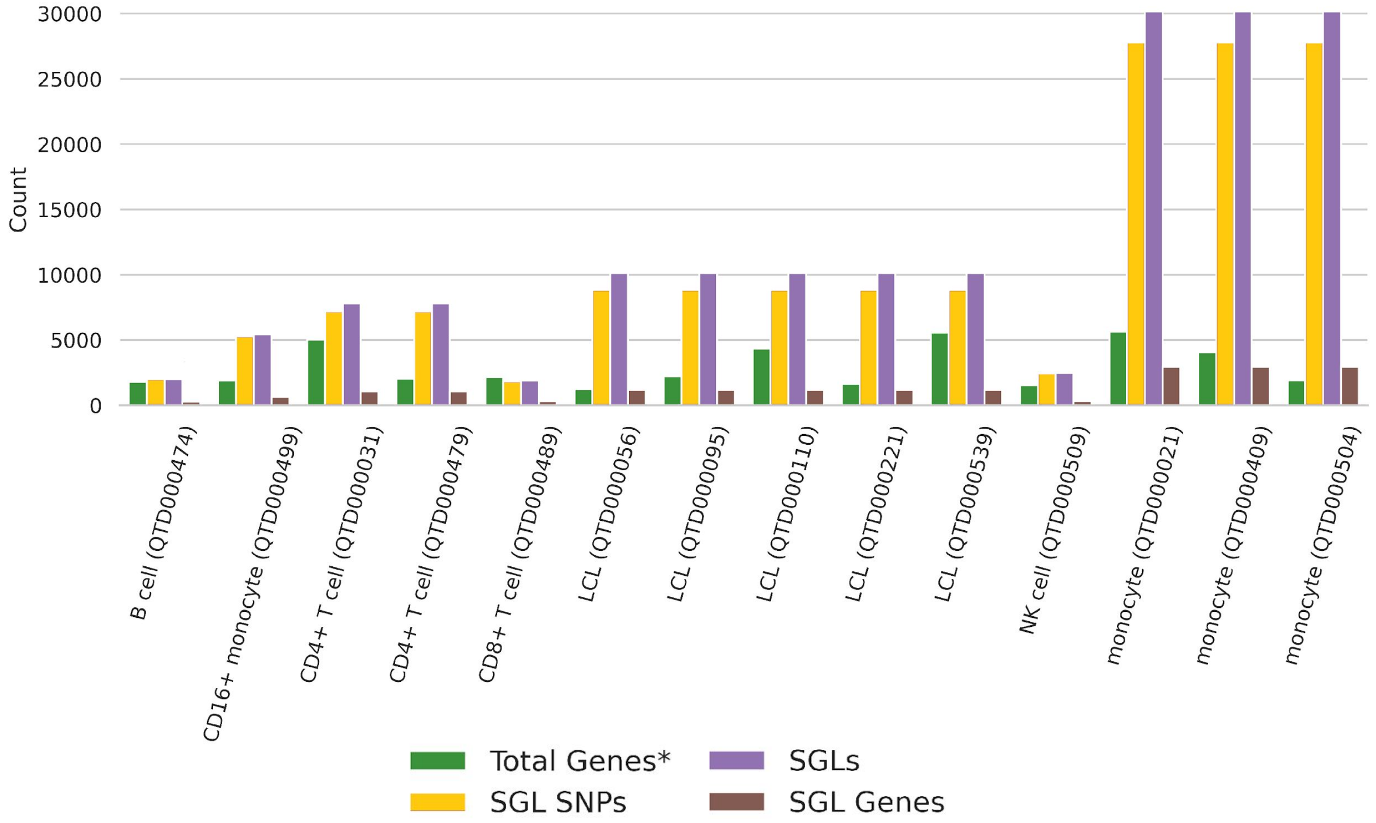

Count
0
5000
10000
15000
20000
25000
30000
B cell (QTD000474)
CD16+ monocyte (QTD000499)
CD4+ T cell (QTD000031)
CD4+ T cell (QTD000479)
CD8+ T cell (QTD000489)
LCL (QTD000056)
LCL (QTD000095)
LCL (QTD000110)
LCL (QTD000221)
LCL (QTD000539)
NK cell (QTD000509)
monocyte (QTD000021)
monocyte (QTD000409)
monocyte (QTD000504)
Total Genes*
SGLs
SGL SNPs
SGL Genes

Fig. S13: Summary of SGLs derived from HiChIP and eQTL intersections. A) Breakdown by cell type for the total number of eQTLs (blue) and SNPs (orange) within the original eQTL dataset. **B)** Breakdown by cell type for the total number of genes from the eQTL study (green) followed by SGL summaries for unique SGLs (yellow), SNPs (purple) and genes (brown). * indicates a total number of SNPs or genes derived from the eQTL study before intersection with HiChIP loops.

Fig. S14

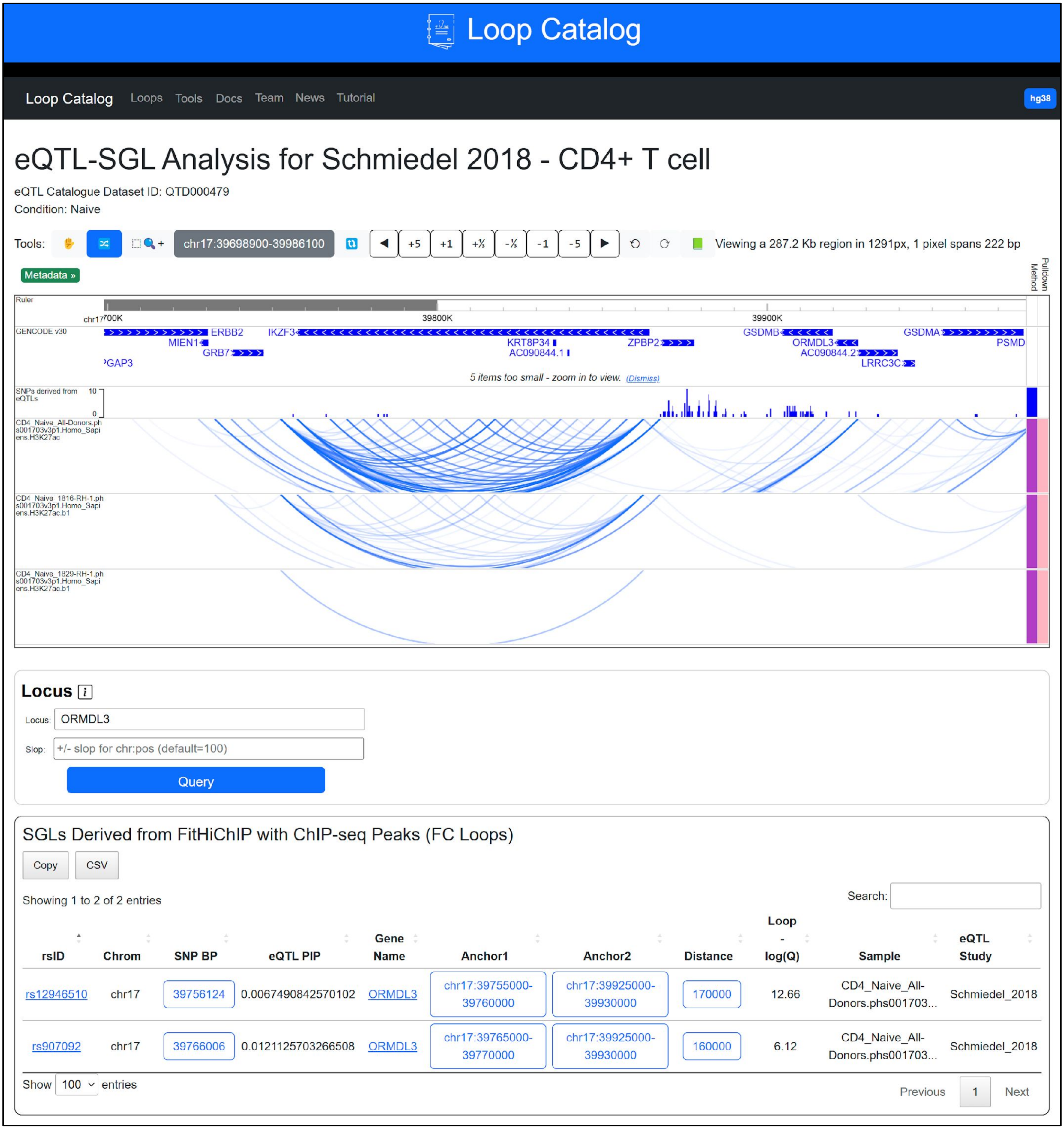
Loop Catalog
Loop Catalog
Loops
Tools
Docs
Team
News
Tutorial
hg38
eQTL-SGL Analysis for Schmiedel 2018 - CD4+ T cell
eQTL Catalogue Dataset ID: QTD000479
Condition: Naive
Tools:
chr17:39698900-39986100
Viewing a 287.2 Kb region in 1291px, 1 pixel spans 222 bp
Metadata »
5 items too small - zoom in to view. (Dismiss)
Locus
Locus: ORMDL3
Slop: +/- slop for chr:pos (default=100)
Query
SGLs Derived from FitHiChIP with ChIP-seq Peaks (FC Loops)
Copy
CSV
Showing 1 to 2 of 2 entries
Search:
rsID | Chrom | SNP BP | eQTL PIP | Gene Name | Anchor1 | Anchor2 | Distance | Loop - log(Q) | Sample | eQTL Study
rs12946510 | chr17 | 39756124 | 0.00674908425701 02 | ORMDL3 | chr17:39755000-39760000 | chr17:39925000-39930000 | 170000 | 12.66 | CD4_Naive_All-Donors.phs001703... | Schmiedel_2018
rs907092 | chr17 | 39766006 | 0.0121125703266508 | ORMDL3 | chr17:39765000-39770000 | chr17:39925000-39930000 | 160000 | 6.12 | CD4_Naive_All-Donors.phs001703... | Schmiedel_2018
Show 100 entries
Previous
1
Next

Fig. S14: Example analysis for CD4+ T cells using SGLs derived from their corresponding eQTLs. Depicted is the IKZF3/ORMDL3 locus with the top grey track containing SNPs derived from eQTLs followed by H3K27ac HiChIP loops for six CD4+ T cell samples.
